# Patterns of Adaptation in Child-Directed and Child Speech in the Emergence of Hebrew Verbs

**DOI:** 10.3389/fpsyg.2021.719657

**Published:** 2021-10-13

**Authors:** Elitzur Dattner, Ronit Levie, Dorit Ravid, Orit Ashkenazi

**Affiliations:** ^1^Department of Communication Disorders, Tel Aviv University, Tel Aviv, Israel; ^2^School of Education, Tel Aviv University, Tel Aviv, Israel; ^3^Department of Communication Disorders, Haddassa College, Jerusalem, Israel

**Keywords:** CS-CDS adaptation, network analysis, Hebrew, roots and patterns, dynamic network analysis

## Abstract

Children approach verb learning in ways that are specific to their native language, given the differential typological organization of verb morphology and lexical semantics. Parent-child interaction is the arena where children's socio-cognitive abilities enable them to track predictive relationships between tokens and extract linguistic generalizations from patterns and regularities in the ambient language. The current study examines how the system of Hebrew verbs develops as a network over time in early childhood, and the dynamic role of input-output adaptation in the network's increasing complexity. Focus is on the morphological components of Hebrew verbs in a dense corpus of two parent-child dyads in natural interaction between the ages 1;8-2;2. The 91-hour corpus contained 371,547 word tokens, 62,824 verb tokens, and 1,410 verb types (lemmas) in CDS and CS together. Network analysis was employed to explore the changing distributions and emergent systematicity of the relations between verb roots and verb patterns. Taking the Semitic root and pattern morphological constructs to represent linked nodes in a network, findings show that children's networks change with age in terms of node degree and node centrality, representing linkage level and construct importance respectively; and in terms of network density, as representing network growth potential. We put forward three main hypotheses followed by findings concerning (i) changes in verb usage through development, (ii) CS adaptation, and (iii) CDS adaptation: First, we show that children go through punctuated development, expressed by their using individual constructs for short periods of time, whereas parents' patterns of usage are more coherent. Second, regarding CS adaptation within a dynamic network system relative to time and CDS, we conclude that children are attuned to their immediate experience consisting of current CDS usage as well as previous usage in the immediate past. Finally, we show that parents (unintentionally) adapt to their children's language knowledge in three ways: First, by relating to their children's current usage. Second, by expanding on previous experience, building upon the usage their children have already been exposed to. And third, we show that when parents experience a limited network in the speech of their children, they provide them with more opportunities to expand their system in future interactions.

## Introduction

Network analysis is increasingly common in various areas of science, from social studies to the spread of epidemics (Kolaczyk, 2009), as it captures relations within the data and allows the statistical assessment of the structure of links between data components (Chen et al., 2018). In linguistics, network analysis has mostly been used to explain the structure and development of semantic networks (Beckage et al., 2011). The present study aims to model the development of Hebrew verb morphology—that is, the system of relations between roots and inflected patterns. We look at patterns of adaptation between Child Speech and Child Directed Speech (van Geert, 1991; van Dijk et al., 2013), expressed in changes within their respective morphological systems. The development of the system is shown to be complex and dynamic, such that attributes of the child's system are affected by other attributes within the system, as well as by the parent's system, and vice versa. In order to account for the verb lexicon morphology as a system, we adopt a network-based framework that allows for measuring complex relations between morphological constructs and their dynamic changes as a function of development and adaptation.

In light of these objectives, the current paper extends linguistic network analysis in two important directions. One is developmental: while language learning makes use of low-level generalizations, taking into account frequency and similarity of exemplars (Ambridge, 2020), the adaptive nature of language development entails the growing complexity of networks (Beckner et al., 2009). A second direction is morphological: Network analysis makes it possible to underscore the role of links between morphemes and the structure that emerges from these connections. The present study utilizes measures of network structure to explain early morphological development of the Hebrew verb system in the context of parent-child interaction and adaptation.

### Input–Output Relations in Language Development

Parent-child interactions constitute the arena in which children use their cognitive and social abilities to extract patterns and regularities from the ambient language. Interactional support, linguistic adaptation and conceptual challenge promote language learning during these interactions (Rowe and Snow, 2020). In the realm of usage-based language acquisition, this type of linguistic input, also termed Child Directed Speech (CDS), is fine-tuned to the child's age and linguistic abilities (Snow, 1995; Ko, 2012). For the child, CDS is the major source of information about the morphology, syntax and semantics of the language being acquired (Hoff-Ginsberg, 1985; Maslen et al., 2004; Behrens, 2006). Usage-based analyses have shown that children detect patterns in the speech they hear and form generalizations by using the socio-cognitive abilities of intention reading, coupled with statistical learning and consequent schematization (Saffran, 2003; Tomasello, 2003, 2006, 2009). Abstract categories gradually emerge out of the items children have learned, based on the distributional and frequency properties of the input (Lieven et al., 2003; Tomasello, 2004; Lieven, 2008).

Focusing on the acquisition and development of verbs, studies on input-output relations have revealed clear correlations between features of verbs in CDS and their realization in Child Speech (CS). These include morphological characteristics of verbs (Acsu-Koc, 1998; Xantos et al., 2011) and their lexical semantics (Montag et al., 2015); syntactic properties of verbs in their environments (Naigles and Hoff-Ginsberg, 1998; Goldberg, 2006; Arunachalam et al., 2011); and their pragmatic features (Cameron-Faulkner, 2012; Clark and de Marneffe, 2012; Ninio, 2014). Of particular interest to the present study is the development of Hebrew verb morphology as a system that develops over time in early childhood, and the role of input-output relations and adaptation in the network's increasing complexity.

Recent studies have shown that Hebrew acquiring toddlers rely on stable, frequently occurring inflectional verb affixes in maternal input to gain salient information on the opaque, irregular verbs they frequently encounter (Ashkenazi et al., 2016). Furthermore, correlations were found between Child Directed Speech and Child Speech in terms of verb lemmas and their morphological components—structural root categories, *binyan* conjugations, and derivational verb families. Clear CDS-CS relations were also found between lexical-derivational development and inflectional growth as measured by Mean Size of Paradigm (MSP; Ashkenazi et al., 2020). The current study delves deeper into the development of morphological complexity in the verb domain by computing developmental changes in root, pattern and inflectional morphology in the dyadic interactions of two toddlers and their respective parents.

### Morphological Constructs in Hebrew Verbs

Three morphological constructs are relevant to the current study: Semitic roots, *binyan* patterns, and subject-verb agreement markers.

#### The Semitic Root Network

The morphological construct termed the Semitic root (e.g., *m-s-r* “deliver,” *g-d-l* “grow”) is a central feature of Semitic languages. This is a (usually) tri-literal consonantal string that constitutes the formal and semantic core of many Hebrew words, and most especially, of all Hebrew verbs (Laks, 2013; Kastner, 2019; Ravid, 2019). Many studies point to the Semitic root as the most accessible Hebrew morpheme in spoken and written language development and usage (Ravid and Bar-On, 2005; Gillis and Ravid, 2006; Schiff et al., 2012; Ben-Zvi and Levie, 2016; Deutsch and Kuperman, 2019), including contexts of language disability or environmental deprivation (Ravid and Schiff, 2006; Schiff and Ravid, 2007; Levie et al., 2017, 2019). Young Hebrew-speaking children demonstrate an early ability to extract roots from familiar words and use them in novel forms (Berman, 1985, 2000, 2012; Ravid, 2003). While a root is not a verb, it functions as a consonantal skeleton shared by different verbs—e.g., *r-d-m* in *nirdam* “fall asleep,” *hirdim* “make sleep;” or *r-g-l* in *hirgil* “make familiar” and *hitragel* “get used”—often carrying a shared basic lexical semantics, creating derivational verb families (Levie et al., 2020). Therefore, roots are key in Hebrew morpho-lexical development as the organizers of root-based networks in the verb lexicon. Within the network-based framework of the current paper, the root is a morphological construct which is conceived as a node in a morphological network.

#### The Semitic *Binyan* Network

As a consonantal, discontinuous entity, the Semitic root is not pronounceable, and as a sub-lexical bound morpheme, it has no lexical category. It is thus always complemented by the Semitic *binyan* (lit. “building”), a prosodic template interspersing root radicals with vowels, often preceded or followed by a small set of pattern affixes, as in *maskim* “agrees,” pattern *maCCiC*. There are seven *binyan* conjugations respectively termed *Qal, Nif'al, Hif'il, Huf'al, Pi'el, Pu'al*, and *Hitpa'el*, which are affixed to roots to create verb lemmas (Schwarzwald, 1981; Berman, 1993a,b; Berman, 2012). For example, *siper* “tell” is expressed by the combination of root *s-p-r* and *binyan Pi'el*; *yarad* “go down” as the combination of root *y-r-d* with *Qal*, and *horid* “take down” as the combination of root *y-r-d* with *Hif'il* (the last two sharing a root, but being two discrete verb lemmas).

In tandem with roots, *binyan*-based conjugations thus constitute networks organizing the Hebrew verb lexicon in morpho-phonological patterns associated with a set of transitivity and Aktionsart functions (Berman, 1993a,b; Kastner, 2016; Ravid, 2019). On the one hand, root-*binyan* verb lemmas form derivational verb families, where verbs with different *binyan* patterns are based on a single shared root (Bolozky, 1999; Ravid, 2019). Consider, for example, the network of verbs sharing root *l-m-d*: *lamad* “learn” (in *Qal*), its passive counterpart *nilmad* “be learned” (*Nif'al*), the causative verb *limed* “teach” (*Pi'el*), and the middle-voice verb *hitlamed* “apprentice” (*Hitpa'el*) (Berman, 1987). From a complementary perspective, verbs with different roots share the same *binyan* conjugation, as demonstrated by the causative verbs *higbir* “make stronger,” *higdil* “make bigger,” *histir* “hide,Tr” and *hiklit* “record,” all sharing the *Hif'il* pattern, with different roots. Similarly to roots, the current framework takes a *binyan* (with temporal patterns and agreement inflections, see below) to be a morphological construct which is conceived as a node in a morphological network.

Note that in young children and parental speech, most verbs are based on *Qal*, the most prevalent *binyan* in Hebrew. With age, children are exposed to larger root-pattern networks that highlight the shared vocalic structure of verbs, making it possible for *binyan* conjugations and their syntactic-semantico values to be learned (Levie et al., 2020). The increase in number, size and complexity of networks of root-related derivational verb families is a clear indicator of a growing verb lexicon (Ravid et al., 2016; Levie et al., 2019).

##### Temporal Patterns Within Binyan Conjugations

In the current framework of analysis, the notion of verb pattern relates the derivational notion of *binyan* to the inflectional paradigm within each *binyan*. Each of the seven conjugations termed *binyanim* actually consists of a phonologically unique bundle of five temporal patterns—past tense, present tense, future tense, imperative, and infinitive forms—as depicted in Table 1. Temporal pattern templates determine the basic morpho-phonology of the verb stem, including root radical slots and vowel combinations. This means that temporal shifts within the same *binyan* paradigm require the use of the same root, each time combining with a different *binyan*-unique temporal pattern. For example, *CaCaC, CoCeC*, and *li-CCoC* (where C's stand for root radicals) serve as the respective past, present and infinitive patterns of *Qal*. When combined with root *k-t-b* “write,” the stems *katav* “wrote,” *kotev* “writes/writing,” and *li-xtov* “to-write” are yielded, respectively. In the same way, *hiCCiC, maCCiC, yaCCiC*, and *le-haCCiC* serve as the respective past, present, future and infinitive patterns of *Hif'il*, combining with *k-t-b* to respectively yield *hixtiv* “dictated,” *maxtiv* “dictates/dictating,” *yaxtiv* “will dictate,” and *le-haxtiv* “to-dictate.” Given the prominence of the root and *binyan* morphemes in the Hebrew lexicon, this process is critical in the acquisition of verb morphology (Berman, 1987; Ravid, 2003).

**Table 1:** The seven *binyan* conjugations as sets of temporal patterns.

***Binyan***	**Past tense**	**Present tense**	**Future tense**	**Imperative**	**Infinitive**
*Qal*	*CaCaC*	*CoCeC*	*yiCCoC*	*CCoC*	*liCCoC*
*Nif'al*	*niCCaC*	*niCCaC*	*yiCaCeC*	*hiCaCeC*	*lehiCaCeC*
*Hif'il*	*hiCCiC*	*maCCiC*	*yaCCiC*	*haCCeC*	*lehaCCiC*
*Huf'al*	*huCCaC*	*muCCaC*	*yuCCaC*	—	—
*Pi'el*	*CiCeC*	*meCaCeC*	*yeCaCeC*	*CaCeC*	*leCaCeC*
*Pu'al*	*CuCaC*	*meCuCaC*	*yeCuCaC*	—	—
*Hitpa'el*	*hitCaCeC*	*mitCaCeC*	*yitCaCeC*	*hitCaCeC*	*lehitCaCeC*

As Table 1 shows, Hebrew speaking children are faced with 31 *binyan*-specific temporal patterns that need to be learned. In the current analysis, when we refer to a *verb pattern*, we actually refer to one of these 31 *binyan*-unique temporal patterns. From a developmental perspective, this construal of verb patterns is a facilitating property of the system, so that for the young child, root-based relations in the verb system can first be learned by attending to the root-pattern temporal shifts within the same *binyan* (Ashkenazi et al., 2016, 2020). Table 1 shows that, while some temporal patterns are phonologically similar (e.g., the temporal paradigm of *Hitpa'el*), others (e.g., those of *Qal* and *Nif'al*) display more phonological distinctions. This is important, as *Qal*, which occupies about 80% of the verb tokens heard or produced by children up to 3 years of age, has the most phonologically distinct temporal patterns, a boost to the transparency-aided acquisition of root and pattern structure (Ravid, 2019).

To illustrate the central role of this network, think about noting the formal resemblance of verbs sharing the *meCaCeC* present-tense *Pi'el* pattern (e.g., *medaber* “talking,” *meshaker* “lying,” *melamed* “teaching”), the similarity of their temporal semantics, and their relation to other *Pi'el* patterns such as past-tense *CiCeC* in *diber* “talked,” *shiker* “lied,” and *limed* “taught” respectively.

Recent research (Ashkenazi et al., 2016, 2020; Ravid et al., 2016) indicates that young Hebrew-speaking children initially learn to manipulate roots and patterns in the inflectional shifts across the temporal stems in the paradigm of a single *binyan* (most often the ubiquitous *Qal*), where semantic coherence of roots is highest. This is in fact the launching pad of non-linear formation in the verb system. Evidence of errors from toddlers and young children acquiring the *binyan*-temporal system indicates that it takes time and linguistic experience for this knowledge to crystallize toward the beginning of elementary school (Berman, 1982; Ravid, 1995). It is only later on, at schoolage, that verb lemmas in different *binyan* conjugations sharing the same root—i.e., derivational families—enrich the young verb lexicon (Levie et al., 2020). The larger, more numerous and varied root-based verb networks in the lexicon of the language learner (both inflectional, across the temporal paradigm of a single *binyan*, and derivational, across different *binyan* conjugations)—the more complex, productive and abstract the organization of the lexical network relying on roots (Levie et al., 2020).

##### Agreement Inflection

The verb stem created by the non-linear affixation of root plus *binyan*-unique temporal pattern is further inflected for number, gender, and person in agreement with the grammatical subject. Unlike temporal shifts, verb agreement inflection is linear, taking the verb stem rather than the root as its base. For example, *nimsor* “we will deliver” is composed of root *m-s-r* in the future tense pattern of *Qal*, with the prefix *n-* designating the first person plural; and *masru* “they delivered” is composed of root *m-s-r* in the past tense pattern of *Qal*, with the suffix *-u* designating the third person plural. Note, however, that the actual formation of a specific verb (wordform) requires morpho-phonological changes in the stem that are typical of each *binyan*, root type and temporal category. This is not investigated in our current study.

Table 2 presents an overview on agreement marking of Hebrew verbs. In general, it shows that the only temporal category which does not require agreement inflection is the infinitive form; and that present tense verbs are marked for number and gender, but not for person agreement.

**Table 2:** Subject-verb agreement in Hebrew verbs.

**Temporal category**	**Person**	**Number**	**Gender**
Infinitive	X	X	X
Imperative	V	V	V
Future tense	V	V	V
Present tense	X	V	V
Past tense	V	V	V

Table 3 presents a detailed view of the 25 pattern-inflection categories identified in Ashkenazi's (2015) corpus, which constitutes the database of the current study. Each category represents a temporal pattern (Infinitive, Imperative, Future tense, Present tense, or Past tense) with all possible agreement marking (e.g., past tense 3rd person plural). The actual examples in Table 3 are the 25 wordforms constituting the temporal category-agreement inflectional paradigm of *Qal* with root *l-q-*ℏ “take.”^1^

**Table 3:** Hebrew verb inflectional categories.

**Coding**	**Inflectional category**	**Example (root *l-q-*ℏ + *Qal*)**
1	Infinitive	*lakáxat'*^a^ “to take”
2	Imperative, masculine, singular	*kax* “take.Masc”
3	Imperative, feminine, singular	*kxi* “take.Fm”
4	Imperative, plural	*kxu* “take.Pl”
5	Future, 1st person, singular	*ekax* “I will take”
6	Future, 2nd person, masculine, singular	*tikax* “you.Masc.Sg will take”
7	Future, 2nd person, feminine, singular	*tikxi* “you.Fm.Sg will take”
8	Future, 3rd person, masculine, singular	*yikax* “he will take”
9	Future, 3rd person, feminine, singular	*tikax* “she will take”
10	Future, 1st person, plural	*nikax* “we will take”
11	Future, 2nd person, plural	*tikxu* “you.Pl will take”
12	Future, 3rd person, plural	*yikxu* “they will take”
13	Present, masculine, singular	*lokéax* “take/s/taking.Masc”
14	Present, feminine, singular	*lokáxat* “take/s/taking.Fm”
15	Present, masculine, plural	*lokxim* “take/taking.Pl”
16	Present, feminine, plural	*lokxot* “take.Pl.Fm”
17	Past, 1st person, singular	*lakáxti* “I took”
18	Past, 2nd person, masculine, singular	*lakáxta* “you.Masc.Sg took”
19	Past, 2nd person, feminine, singular	*lakaxt* “you.Fm.Sg took”
20	Past, 3rd person, masculine, singular	*lakax* “he took”
21	Past, 3rd person, feminine, singular	*lakxa* “she took”
22	Past, 1st person, plural	*lakáxnu* “we took”
23	Past, 2nd person, masculine, plural	*lakáxtem* “you.Masc.Pl took”
24	Past, 2nd person, feminine, plural	*lakáxten* “you.Fm.Pl took”
25	Past, 3rd person, plural	*lakxu* “they took”

a *Stress in Hebrew is usually final, thus it is only marked if penultimate*.

#### The Current Research

Against this background, the present study has two main objectives: (i) to model the systematic development of the morphology of the Hebrew verb lexicon—that is, the system of relations between roots and inflected patterns; and (ii) to account for various patterns of adaptation between Child Speech and Child Directed Speech (van Geert, 1991; van Dijk et al., 2013), expressed in changes within their respective morphological system structures. The development of the system is shown to be adaptive and complex, such that attributes of the child's system are affected by other attributes within the system, as well as by the parent's system structure, and vice versa. Both the systematic development and the patterns of adaptation are shown to be dynamic, in that the system's structure at one point in time affects its structure in the future. In order to account for verb morphology as a dynamic system, we adopt a network-based framework that allows for measuring complex relations between morphological constructs and their dynamic changes as a function of development and adaptation.

A Dynamic Network Model assumes that higher-order properties are emergent phenomena, such that structure emerges on the basis of the dynamic interactions between lower-level components (Barabasi, 2009; Den Hartigh et al., 2016). This view is compatible with recent usage-based approaches to cognitive representation of language, in which learning is construed as constantly updating connection weights between nodes based on experience (Bybee and McClelland, 2005; Kapatsinski, 2018). The morphological network of the verb lexicon is dynamic in the sense that the values of the constructs it comprises change as a consequence of the interactions with other morphological constructs (among other factors). For example, the importance of a particular root within the verb lexicon can be affected by the importance of the pattern(s) it is linked to (creating specific verb wordforms). Thus, if a low frequency root is linked to a low frequency pattern, it may have consequences for the entrenchment of the verb wordform within cognitive representation, and thus for future usage.

Dynamic network analysis can be helpful in accounting for another facet of dynamicity: over developmental time, morphological constructs may appear or disappear; it is not the case that we use every single root, *binyan* temporal pattern and agreement inflection in our lexicon every single day. Treating development as a dynamically changing set of networks enables us to evaluate such punctuated growth, accounting for accumulated change. For example, the probability of using a particular pattern on a particular day may be higher if that pattern was used the day before (either by the child or by the parent) than if it was not. In the following section we present our data and methods for constructing the network and modeling development.

## Data and Method

### Data

The analyses reported below are based on a densely recorded corpus of naturalistic longitudinal interactions of two Hebrew-speaking parent-child dyads—a boy dyad and a girl dyad. The boy dyad was recorded between the ages 1;8.27 (1 year 8 months and 27 days, or 635 days) to 2;2.3 (2 years 2 months and 3 days, or 795 days), yielding 49 recording sessions. The girl dyad was recorded between the ages of 1;9.25 (1 year 9 months and 25 days, or 664 days) to 2;2.19 (2 years 2 months and 19 days, or 810 days), yielding 47 recording sessions. Different child genders were chosen so as to permit analysis of the obligatory gender agreement in Hebrew verb inflection (Schwarzwald, 1998; Ravid and Schiff, 2015). Both families were from mid-high SES background, living in central Israel. The two sets of parents, who did not know each other, were monolingual native-born speakers of Hebrew. They did not receive any monetary remuneration for their voluntary participation.

Both children were first-born and had no siblings at the time of recording. Both had normal cognitive, communicative, and linguistic development according to parental report (including the Hebrew CDI checklist in Maital et al., 2000), periodic assessment at the local neonate and children's health clinic, and assessment by the last author (a certified senior SLP). Neither of them had a history of ear infections or any other major health issues. The boy attended nursery school and the girl did not. Table 4 summarizes the corpus details (Ashkenazi, 2015).

**Table 4:** The corpus details.

		**Girl (Child 1)**	**Boy (Child 2)**
Age range		1;9.25-2;2.19	1;8.27-2;2.3
# recordings		47	49
Word tokens	CS	39,717	32,369
	CDS	158,679	140,782
Verb types	CS	204	172
= lemmas	CDS	531	503
Verb tokens	CS	4,610	3,101
	CDS	31,283	23,830

#### Data Collection

The children were audio- and video recorded by their parents at home during bath time, play time and meal time using an MP3 recorder and a video camera supplied to the family. Each dyad was audio recorded twice a week and video recorded once a week, for 45–60 min each time, for 6 months between 1;8-2;2 approximately (see details above). The parents were informed that the study concerned early language development in Hebrew. They were asked to record spontaneous, natural interactions. Recordings of both dyads started when each child started producing two word utterances and some verbs, based on parental reports using the Hebrew CDI (Maital et al., 2000). Transcriptions of the recordings (see below) ceased when each child produced subject-verb agreement in number and gender in two subsequent recordings, including two different person agreements in past tense, on at least two different verbs. This morphosyntactic criterion indicated that the child was gaining command of the basic components of verb structure and semantics by productively using temporal stems, that is, root-pattern alternations, as well as agreement markers (Berman and Lustigman, 2012; Ravid et al., 2016). All interactions were coded and analyzed, including nursery rhymes and songs in the parental input, as well as speech addressed to the other parent (which consisted <5% the recordings).

#### Transcription

Dyadic interactions were transcribed in broad phonemic transcription following the CHILDES conventions (MacWhinney, 2005), adapted to take into account Hebrew-specific phonemic, phonological, prosodic, and orthographic features (Albert et al., 2013). The transcriptions were carried out by undergraduate students of an academic SLP program who took a CHILDES course as part of their studies. The recordings were thoroughly checked by the last author and corrected when necessary, with an estimated 5% error rate. Next Hebrew MOR was run over the transcripts. Ambiguous forms and verb forms that were not analyzed by the program were identified and coded manually.

### Method

#### Morphological Variables

**Figure 1:**
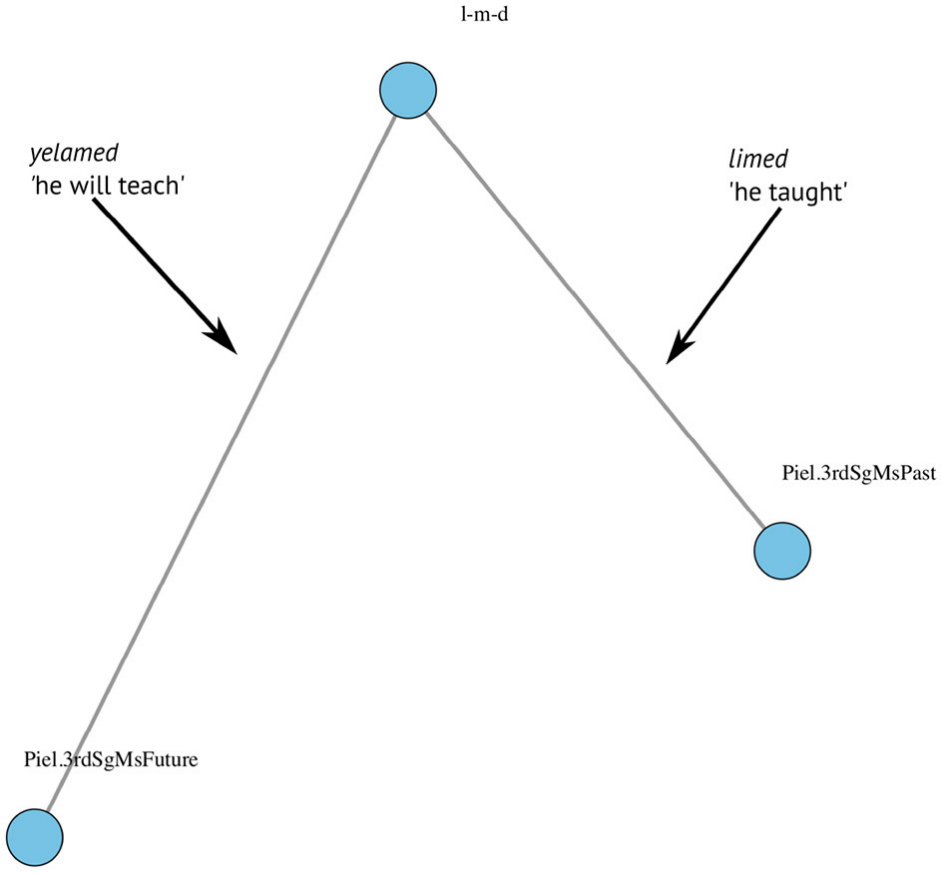
A Hebrew morphological verb network: an illustration. The root *l-m-d* is linked to two *binyan* temporal-inflection patterns, yielding two verb wordforms: *l-m-d*+*Piel*.3rdSgMsPast is *limed* “He taught,” and *l-m-d*+*Piel*.3rdSgMsFuture is *yelamed* “He will teach”.

Three variables participated in the network analysis described below:

1. Root: The Semitic consonantal construct at the basis of the Hebrew verb, e.g., *s-y-m* “put,” *z-h-r* “take care,” *r-d-m* “sleep,” or *n-g-b* “towel.”
2. Pattern + Agreement: This was the complementary construct to the root. In the current analysis, it consisted of (i) the *binyan*-specific temporal pattern (see Table 1 for the full array of *binyan*-temporal patterns); and (ii) the person-number-gender agreement inflection (see Table 3 for the full array of agreement inflections). Note that 1 and 2 are the two morphological constructs that participate in the verb structure, rather than actual words.
3. Verb wordform: The *actual* word as appearing in the transcription: a unique combination of a root and a *binyan*-temporal pattern + agreement marking, as in the following four examples (see also Figure 1):
  1. *sámti* “I put” = *s-y-m* + *Qal* past tense, 1st Sg;
  2. *tizahari* “take care!” = *z-h-r* + *Nif'al* imperative, 2nd Sg Fm;
  3. *nirdamim* “are falling asleep” = *r-d-m* + *Nif'al* present tense, Pl Masc;
  4. *yitnagev* “(he will) towel (himself)” = *n-g-b* + *Hitpa'el* future tense, 3rd Sg Masc.

#### Network Analysis

For each child and parent in the data we constructed a list of all available roots and inflected patterns throughout the entire database, resulting in four lists. These lists formed the basis for the network analysis, such that each participant used a subset of their list on a particular recording. The nodes of the bipartite network of recording *N* are the list of roots and inflected *binyan* patterns that appear in recording *N*, creating links that stand for the actual verb wordforms that were used in recording *N* by participant X. For example, a token of the root *l-m-d* and the inflected pattern <*Pi'el*, masculine, singular, third person, past tense> constitutes one link yielding the wordform *limed* “taught.3.Sg.Ms.”; while a token of the same root (*l-m-d*) and the inflected pattern <*Pi'el*, masculine, singular, third person, future tense> constitutes another link, yielding the wordform *yelamed* “will teach.3.Sg.Ms.” That is, verb wordforms are *links* between *nodes* in a morphological *network*, as illustrated in Figure 1.

##### Node Level Measure: Degree

*Node degree* is a centrality measure, corresponding to the number of links a node has with other nodes in a network. Degree (*C*_*D*_) is calculated as:


(1) $$\begin{array}{l} C_{D}\left(j\right)\;=\;\overset{n}{\underset{j=1}{\sum }}A_{ij} \end{array}$$

for every node in the data, over its corresponding rows and columns of the matrix *A*.

A node with a high degree value is more important in the network as it participates in more language events. *Degree* corresponds to the token frequency of each construct. Thus, a network with a few high degree nodes indicates repeated use of particular types, suggesting a less varied network. We hypothesize that the degree level of nodes will increase with age in the CS, and that degree distribution within the CS networks will change with age, indicating usage variation. These changes are not hypothesized to occur in the CDS networks.

##### Node Level Measure: Eigenvector Centrality

A second centrality measure used here is the *eigenvector centrality* of particular nodes - roots or inflected patterns in our case. To achieve a relevant explanatory assessment of the data, we focus here on eigenvector centrality as reflecting the node's importance (Bonacich, 2007; Lohmann et al., 2010; Oldham et al., 2019). Eigenvector centrality *x*_*i*_ of node *i* is given by:


(2) $$\begin{array}{l} x_{i}\;=\;\frac{1}{\lambda }\underset{k}{\sum }a_{k,i}x_{k} \end{array}$$

where λ ≠ 0 is a constant, and *k* is the node's degree.

A node with high centrality is linked to many other nodes that, in turn, are linked to many other nodes. In non-directed networks, as in the present study, such nodes are said to be in a central, prominent position. For example, an inflected pattern linked to two roots that are themselves linked to three inflected patterns each is higher in centrality than an inflected pattern linked to two roots that are not linked to other patterns. *Centrality* quantifies the significance of a node relative to other nodes in the network. For example, centrality can reveal those morphological patterns that act as centers of gravity for forming verbs, and changes in centrality of a particular pattern can be measured during development. We hypothesize that nodes' centrality will change through development in a non-linear manner, reflecting changes in discourse circumstances, in both the CS and CDS networks. Crucially, these changes are not a matter of mere frequency, but rather of the frequency of links with other frequent nodes.

##### Network Level Measure: Density

While degree and centrality are measures concerning attributes of the *nodes* of the network, the *density* measure concerns the network as a whole. The density of the network is a measure of fulfilled links between nodes (Wasserman and Faust, 1994). Density is a mathematical notion that measures the proportion of observed links relative to the maximum number of possible links: the closer it is to one, the more possible links are actually manifested, and thus the more interconnected the network. Network density (*d*) is calculated as:


(3) $$\begin{array}{l} d\;=\;\frac{m}{n\left(n-1\right)/2} \end{array}$$

where *m* is the total number of existing links in the network, and *n* is the number of nodes in the network. Links within a dense network are more predicted and anticipated. As such, somewhat counter-intuitively, a *sparse* network is taken here to indicate a higher level of potential productivity: In a sparse network, there are more root- and pattern-nodes which are not linked to each other, compared to a dense network in which most of the nodes are already linked. Hence, the potential to link two nodes that have not been linked before, thus creating new verb wordforms, is *higher* in a sparse network, compared to a dense network (Levie et al., 2019). That is, a sparse network means that the pool from which one can choose how to put experience into words, specifically verbs (by linking a root and a pattern) is not exhausted, and new verb wordforms can be created: new links between roots and inflected patterns, which refer to more fine-grained aspects of experience. We hypothesize that network density will decrease with age within the children's networks, but will remain steady through time in the parents' networks.

##### Network Construction and Model Design

For every recording we calculated two networks, one for each participant. This resulted in 94 networks for Child 1 [47 recordings ^*^ (CS + CDS)], and 98 networks for Child 2 [49 recordings ^*^ (CS + CDS)]. We account for these networks as consecutive points in a dynamically evolving network, analyzing the development of network measures as obtained in each instance of network. The three measures presented above were extracted for each network, resulting with a time series data of network density for every participant, the changing degree of each node in the networks through development, and the changing centrality of each node through development.

In order to find patterns of adaptation in network structure as representing the verb lexicon, we assessed the development of network measures for each child and parent separately, and modeled the effect of the child's age on each measure, the effect of CDS network measures on CS network measures, and vice versa. Moreover, since time related data are available, we added to the models the level of each measure in the preceding recording, enabling further assessment of adaptation. For example, we could ask whether the density level of the child's network in the preceding recording affects the parent's level of density in the network of the current recording.

Furthermore, for each node that appeared on a particular day in both the child's and the parent's networks, we modeled its degree and centrality (in CS and CDS, in Child 1 and Child 2, separately) as a function of the other measures in the same recording, as well as the levels of the other measures in the preceding recording. For example, we could ask whether the chance of a root or inflected pattern produced by the child to have high centrality is higher if this root or inflected pattern is central in the parent's network in the preceding recording, and/or is central in the child's preceding recording, and/or has a high degree level in the current recording.

Table 5 summarizes the variables and measures in the study that were part of either the construction of the networks or the model design in analyzing adaptation through development. Each morphological/situational variable and network measure is a part of the four participants design: CS of Child 1, CDS of Child 1, CS of Child 2, and CDS of Child 2. Consequently, the results reported below present four models for each measure. All resulting measurements of the network analysis were centered and scaled before model calculations.

**Table 5:** Summary of study variables.

**Variable**	**Interpretation**
**Situational variables**	
Age	Child's age
Speaker	Child Speech (CS) vs. Child Directed Speech (CDS)
**Morphological variables**	
Verb root	A node in the network
Verb inflected pattern	A node in the network
Verb wordform	A link in the network (linking a root and a pattern)
**Network measures**	
degree.cs	CS node degree at recording N
prior.degree.cs	CS node degree at recording N-1
degree.cds	CDS node degree at recording N
prior.degree.cds	CDS node degree at recording N-1
centrality.cs	CS node centrality at recording N
prior.centrality.cs	CS node centrality at recording N-1
centrality.cds	CDS node centrality at recording N
prior.centrality.cds	CDS node centrality at recording N-1
density.cs	CS network density at recording N
prior.density.cs	CS network density at recording N-1
density.cds	CDS network density at recording N
prior.density.cds	CDS network density at recording N-1

## Results

### Overall Outlook

**Figure 2:**
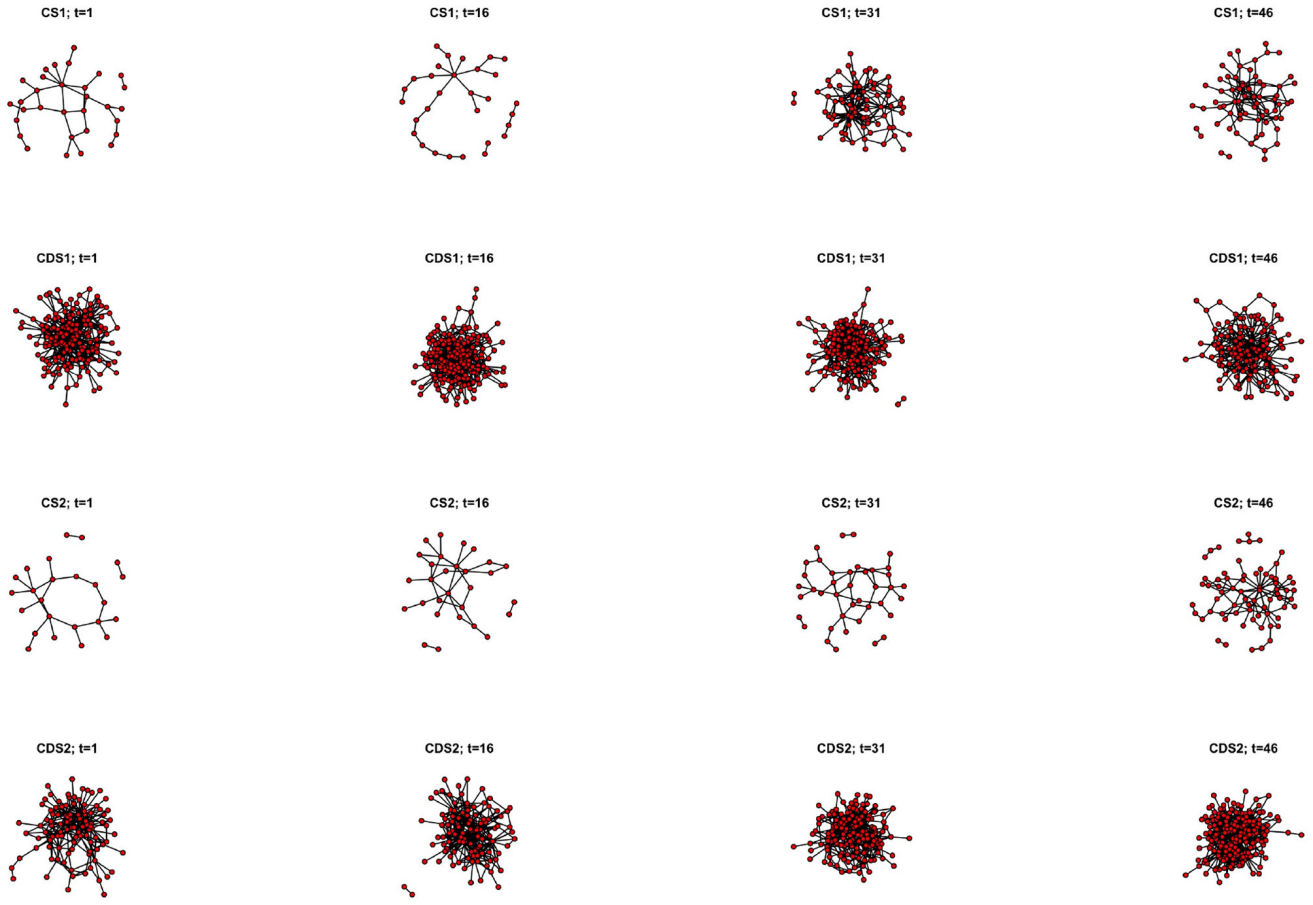
Dynamic view of a developing verb lexicon as a network. Top row: CS of child 1; second row: CDS of child 1; third row: CS of child 2; bottom row: CDS of child 2. Each row portrays four points in the longitudinal corpus: recordings no. 1 (age 1;9.25 for Child 1, 1;8.27 for Child 2); 16 (age 1;11.6 for Child 1, 1;10.7 for Child 2); 31 (age 2;1.1 for Child 1, 2;0.0 for Child 2); and 46 (age 2;2.17 for Child 1, 2;1.23 for Child 2). The nodes of the networks are roots and inflected patterns. Links between nodes represent verb wordforms.

We start off the presentation of our results with an overall outlook on the four changing temporal networks (two networks of children's speech, CS 1 and CS 2; and two networks of Child Directed Speech, CDS 1 and CDS 2), from a dynamic perspective that underscores the emergence of the system. These networks are shown in Figure 2 through four representative time points within the longitudinal corpus: recordings no. 1 (age 1;9.25 for Child 1, 1;8.27 for Child 2); 16 (age 1;11.6 for Child 1, 1;10.7 for Child 2); 31 (age 2;1.1 for Child 1, 2;0.0 for Child 2); and 46 (age 2;2.17 for Child 1, 2;1.23 for Child 2). The nodes of the networks are roots and inflected patterns. Links between nodes represent verb wordforms.

**Figure 3:**
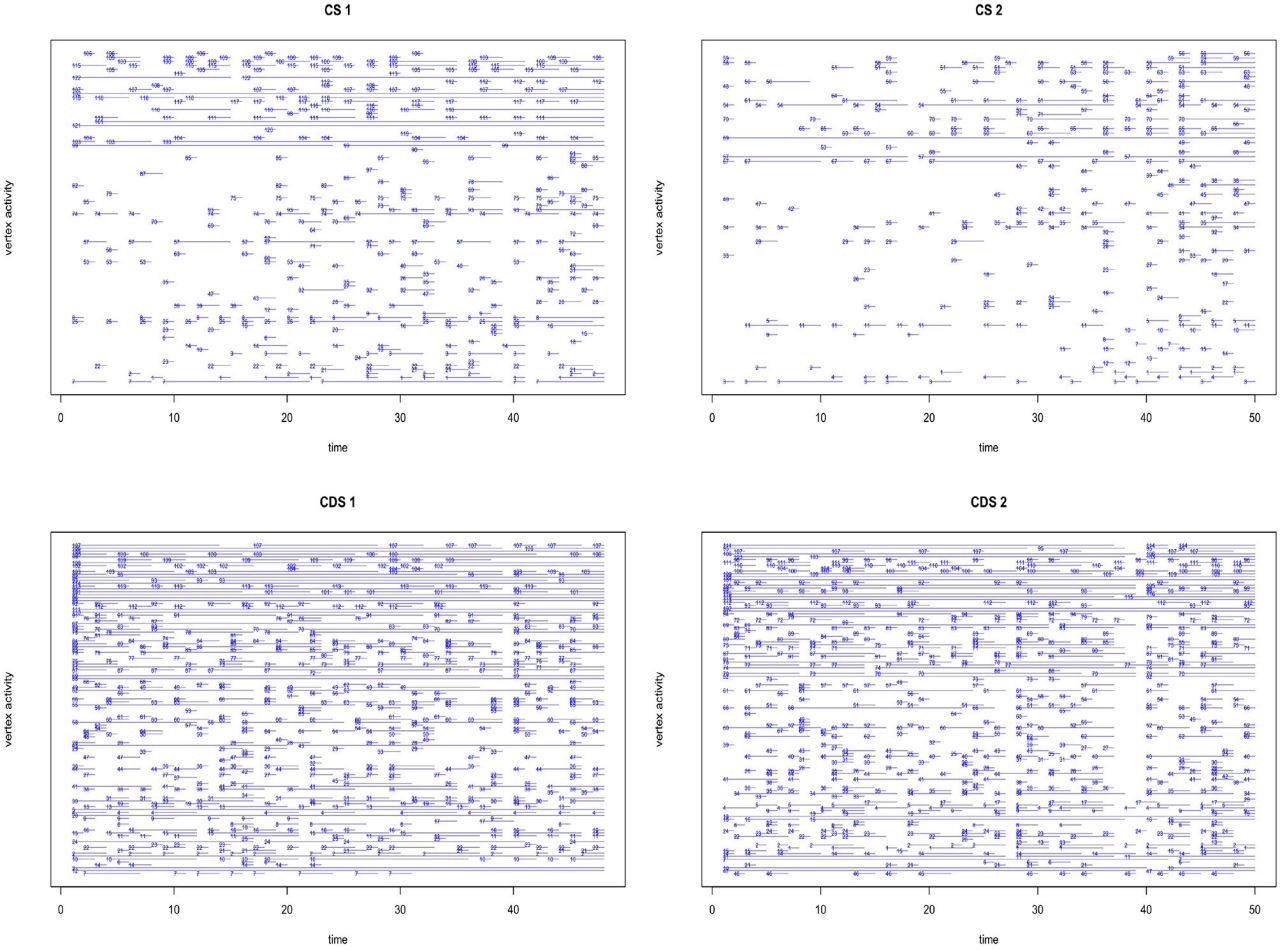
Inflected patterns activity throughout the entire time range in the data. Each line represents the time in which a node is active in the network. Nodes are represented by the numbers at the beginning of each line (root nodes have been removed in order to increase readability).

Figure 2 shows that the children's networks go through much more development than the parents' networks, such that more nodes and more links between nodes are shown with time. That is, in morphological terms, we see growth in the number of roots and inflected patterns, and growth in the number of verb wordforms (cf. Ashkenazi, 2015). Growth in number of nodes and links renders a more complex network, as can be seen by the complex structure of the children's networks in older ages. Moreover, we can see that the structures of the parents' networks remain similar throughout the data, such that it is very complex from the very beginning. The view presented by Figure 2 allows us to observe growth in complexity in a visual manner. The models presented below will add to this view, relating system development to multiple factors. However, before turning to the models results, let us emphasize another facet of dynamic network analysis, that of node activation, as shown in Figure 3.

Morphological constructs in a dynamic perspective are portrayed according to their activation patterns. For example, a link between a root and an inflected pattern may appear in recording number 6 (i.e., the link is active), be absent from recording number 7 (i.e., the link is inactive), and reappear in recording number 8. Figure 3 portrays a timeline of inflected pattern activation throughout development (in the age ranges of the current corpus; root nodes are not represented in order to increase readability of the plot). We can see that the children's networks are characterized by what we term *punctuated development*, such that most of the inflected patterns appear and disappear frequently; while the parents' networks are characterized by more continuous usage of the full array of inflected patterns. This characterization of the development of the morphological system is made possible by the framework of dynamic network analysis, and we will return to its implications in the discussion section below. We now turn to the results of the models. First the two node-level measures (degree and centrality), and then the global network measure (density).

### Node Level Measure: Degree

**Figure 4:**
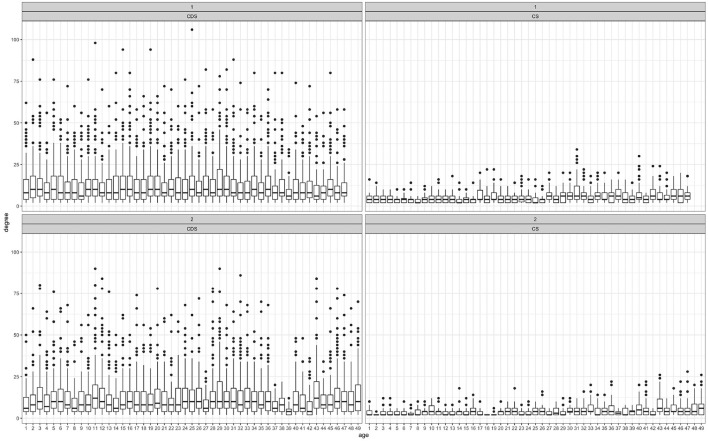
Node degree distribution by session: CS (right panels) and CDS (left panels), child 1 (top panels) and child 2 (bottom panels).

Recall that the degree of an inflected pattern within a network is the number of roots linked with it, and the degree of a root within a network is the number of inflected patterns it is linked to. Figure 4 presents degree distribution through development. Every recording session (the X axis) is a single network within the entire set of networks through time. Each bar represents the degree distribution within a single network as a single point in time.

Figure 4 shows that CDS degree levels are much higher than CS degree levels in both sub-corpora, and that degree level in CS seems to increase with age in both children. That is, parents tend to link more roots to more inflected patterns, and more inflected patterns to more roots, compared with children's linkage distribution.

In order to assess development and adaptation relative to node degree, we fitted our models only on those nodes that appeared in both the child's and the parent's network. Thus, for each participant we fitted a linear mixed model (estimated using REML and nloptwrap optimizer) to predict degree level with the following variables: age, degree of the other party in the dyad in the same recording, and the degrees of both parties of the dyad in the previous recording. We also included eigenvector centrality measures of both parties in current and antecedent networks, and the same for density measures, in order to reveal complex relations within the system and to account for adaptation across time. Each model included the specific root or inflected pattern node as a random effect (coded as *name* in the models below). Standardized parameters were obtained by fitting the model on a standardized version of the dataset. Ninety-five percent Confidence Intervals (CIs) and p-values were computed using the Wald approximation.

#### CS Node Degree

Table 6 shows the results for the linear mixed models for both children, predicting CS degree level. Each model is detailed below.

**Table 6:** Linear mixed model: CS node degree.

**Predictors**	**degree.cs: child 1**		***p***	**degree.cs: child 2**		***p***
	**Estimates**	**CI**		**Estimates**	**CI**	
(Intercept)	−0.73	−0.87 –−0.59	<0.001	−0.7	−0.84 –−0.56	<0.001
age	0.01	0.01 – 0.01	<0.001	0.01	0.00 – 0.01	<0.001
degree.cds	0.07	0.04 – 0.10	<0.001	0.08	0.03 – 0.12	<0.001
prior.degree.cds	0.03	0.00 – 0.06	0.043	−0.01	−0.04 – 0.02	0.566
prior.degree.cs	0.23	0.16 – 0.30	<0.001	0.24	0.15 – 0.32	<0.001
centrality.cs	0.11	0.10 – 0.13	<0.001	0.05	0.03 – 0.06	<0.001
prior.centrality.cs	−0.01	−0.03 – 0.01	0.277	0.01	−0.00 – 0.02	0.241
centrality.cds	−0.03	−0.15 – 0.08	0.596	−0.07	−0.20 – 0.07	0.331
prior.centrality.cds	−0.06	−0.17 – 0.05	0.259	0.07	−0.01 – 0.14	0.078
density.cs	−0.06	−0.10 –−0.03	<0.001	−0.04	−0.06 –−0.02	<0.001
prior.density.cs	0.05	0.01 – 0.10	0.024	0.01	−0.01 – 0.03	0.213
density.cds	0.03	−0.06 – 0.11	0.555	0.04	−0.03 – 0.11	0.28
prior.density.cds	−0.07	−0.16 – 0.02	0.123	−0.1	−0.17 – −0.03	0.005
**Random Effects**						
σ2	0.08			0.08		
τ00	0.01 name			0.01 name		
ICC	0.1			0.15		
*N*	147 name			109 name		
Observations	841			541		
Marg.*R*^2^/Cond.*R*^2^	0.502/0.551			0.396/0.486		

##### CS Node Degree: Child 1

The model's total explanatory power is substantial (conditional *R*^2^ = 0.55) and the part related to the fixed effects alone (marginal *R*^2^) is of 0.50. Within this model, the following variables have a significant effect on CS1 degree level: child's age (positive effect), CDS degree level (positive effect), CDS degree level in recording N-1 (positive effect), CS degree level in recording N-1 (positive effect), CS centrality level (positive effect), CS density (negative effect), and CS density in recording N-1 (positive effect).

##### CS Node Degree: Child 2

The model's total explanatory power is substantial (conditional *R*^2^ = 0.49) and the part related to the fixed effects alone (marginal *R*^2^) is of 0.40. Within this model, the following variables have a significant effect on CS2 degree level: age (positive effect), CDS degree level (positive effect), CS degree level in recording N-1 (positive effect), CS centrality level (positive effect), CS density (negative effect), and CDS density in recording N-1 (negative effect).

#### CDS Node Degree

Table 7 shows the results for the linear mixed models for both parents, predicting CDS degree levels. Each model is detailed below.

**Table 7:** Linear mixed model: CDS node degree.

**Predictors**	**degree.cds: child 1**		***p***	**degree.cds: child 2**		***p***
	**Estimates**	**CI**		**Estimates**	**CI**	
(Intercept)	0.59	0.27 – 0.91	<0.001	0.34	0.02 – 0.65	0.038
age	−0.01	−0.01 –−0.00	0.001	0.01	0.00 – 0.01	0.026
degree.cs	0.32	0.18 – 0.46	<0.001	0.32	0.15 – 0.50	<0.001
prior.degree.cs	−0.13	−0.29 – 0.02	0.091	−0.04	−0.21 – 0.14	0.682
prior.degree.cds	0.14	0.07 – 0.20	<0.001	0.2	0.14 – 0.27	<0.001
centrality.cs	0	−0.04 – 0.04	0.979	−0.01	−0.04 – 0.01	0.304
prior.centrality.cs	0.01	−0.03 – 0.05	0.546	−0.02	−0.04 – 0.00	0.095
centrality.cds	1.84	1.63 – 2.06	<0.001	1.7	1.47 – 1.93	<0.001
prior.centrality.cds	0.19	−0.04 – 0.42	0.102	0.18	0.02 – 0.33	0.024
density.cs	−0.07	−0.13 – 0.00	0.051	−0.01	−0.04 – 0.03	0.713
prior.density.cs	0.14	0.04 – 0.24	0.005	0.09	0.06 – 0.12	<0.001
density.cds	−0.57	−0.75 –−0.39	<0.001	−0.58	−0.71 –−0.45	<0.001
prior.density.cds	−0.36	−0.55 –−0.17	<0.001	−0.3	−0.44 –−0.16	<0.001
**Random Effects**						
σ2	0.35			0.33		
τ00	0.11 name			0.08 name		
ICC	0.24			0.2		
*N*	147 name			109 name		
Observations	841			541		
Marg.*R*^2^/Cond.*R*^2^	0.711/0.781			0.707/0.766		

##### CDS Node Degree: Child 1

The model's total explanatory power is substantial (conditional *R*^2^ = 0.78) and the part related to the fixed effects alone (marginal *R*^2^) is of 0.71. Within this model, the following variables have a significant effect on CDS1 degree level: age (negative effect), CS degree (positive effect), CDS degree in recording N-1 (positive effect), CDS centrality level (positive effect), CS density in recording N-1 (positive effect), CDS density (negative effect), and CDS density in recording N-1 (negative effect).

##### CDS Node Degree: Child 2

The model's total explanatory power is substantial (conditional *R*^2^ = 0.77) and the part related to the fixed effects alone (marginal *R*^2^) is of 0.71. Within this model, the following variables have a significant effect on CDS2 degree level: age (positive effect), CS degree (positive effect), CDS degree level in recording N-1 (positive effect), CDS centrality (positive effect), CDS centrality in recording N-1 (positive effect), CS density in recording N-1 (positive effect), CDS density (negative effect), and CDS density in recording N-1 (negative effect).

### Node Eigenvector Centrality

**Figure 5:**
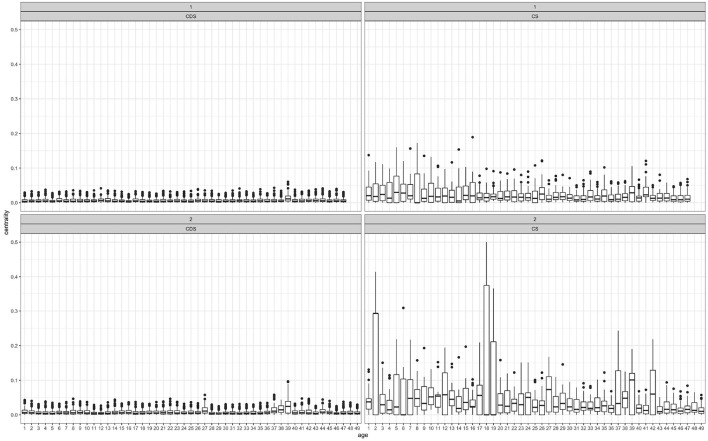
Node centrality distribution by session: CS (right panels) and CDS (left panels), child 1 (top panels) and child 2 (bottom panels).

Recall that the eigenvector centrality of a node is a measure of importance. For example, an inflected pattern has high centrality if it is linked to many roots that are linked to other inflected patterns, that are linked to other roots in turn. Figure 5 presents centrality distribution by recording day, showing a mirror image of degree distribution (Figure 4): Centrality levels within CS networks are much higher than CDS networks. That is, there are more central nodes within the children's network than there are within the parents' networks, and trends in centrality changes are less apparent in the CDS than in the CS.

The models summarized in Tables 8, 9 portray the development of node eigenvector centrality. In a similar model design for the one presented for node degree, we fitted a linear mixed model for each participant (estimated using REML and nloptwrap optimizer) to predict eigenvector centrality with age, centrality of the other party in the dyad in the same recording, and the centralities of both parties of the dyad in the previous recording. We also included degree values of both parties in current and antecedent networks, and the same for density values. Each model included the specific root or inflected pattern node as a random effect (coded as *name* in the models below). Standardized parameters were obtained by fitting the model on a standardized version of the dataset. Ninety-five percent Confidence Intervals (CIs) and *p*-values were computed using the Wald approximation.

**Table 8:** Linear mixed model: CS node centrality.

**Predictors**	**centrality.cs: child 1**		***p***	**centrality.cs: child 2**		***p***
	**Estimates**	**CI**		**Estimates**	**CI**	
(Intercept)	1.58	1.08 – 2.09	<0.001	2.71	1.83 – 3.60	<0.001
age	−0.02	−0.03 –−0.01	<0.001	−0.03	−0.05 –−0.01	0.001
centrality.cds	0.56	0.16 – 0.95	0.006	1.29	0.45 – 2.12	0.003
prior.centrality.cs	0.16	0.09 – 0.22	<0.001	−0.01	−0.08 – 0.07	0.842
prior.centrality.cds	0.39	0.02 – 0.77	0.04	−0.13	−0.66 – 0.39	0.612
degree.cs	1.48	1.26 – 1.70	<0.001	2.68	2.22 – 3.15	<0.001
prior.degree.cs	−0.23	−0.47 – 0.02	0.074			
degree.cds	0	−0.11 – 0.11	0.967	−0.2	−0.47 – 0.08	0.156
prior.degree.cds	−0.08	−0.18 – 0.03	0.146	−0.13	−0.36 – 0.10	0.262
density.cs	0.26	0.15 – 0.37	<0.001	0.6	0.50 – 0.71	<0.001
prior.density.cs	−0.16	−0.33 – 0.01	0.058	−0.06	−0.16 – 0.05	0.304
density.cds	−0.18	−0.48 – 0.13	0.26	−0.58	−1.05 –−0.12	0.014
prior.density.cds	0	−0.32 – 0.32	0.995	0.34	−0.14 – 0.82	0.162
**Random Effects**						
σ2	1.05			4.14		
τ00	0.04 name			0.0 name		
ICC	0.04			0.0		
*N*	147 name			109 name		
Observations	841			541		
Marg.*R*^2^/Cond.*R*^2^	0.442/0.462			0.435/0.435		

**Table 9:** Linear mixed model: CDS node centrality.

**Predictors**	**centrality.cds: child 1**		***p***	**centrality.cds: child 2**		***p***
	**Estimates**	**CI**		**Estimates**	**CI**	
(Intercept)	−0.09	−0.17 – 0.00	0.058	−0.01	−0.11 – 0.10	0.909
age	0	−0.00 – 0.00	0.093	0	−0.00 –−0.00	0.002
centrality.cs	0.01	0.00 – 0.02	0.01	0.01	0.00 – 0.02	0.031
prior.centrality.cs	0	−0.01 – 0.01	0.45	0.01	0.00 – 0.02	0.014
prior.centrality.cds	0.21	0.15 – 0.27	<0.001	0.1	0.05 – 0.14	<0.001
degree.cs	−0.01	−0.05 – 0.03	0.666	−0.02	−0.08 – 0.03	0.374
prior.degree.cs	0.07	0.03 – 0.11	0.001	0.03	−0.03 – 0.08	0.344
degree.cds	0.13	0.11 – 0.14	<0.001	0.15	0.13 – 0.17	<0.001
prior.degree.cds	0	−0.01 – 0.02	0.601	0.03	0.01 – 0.05	0.006
density.cs	0.02	0.00 – 0.04	0.02	0	−0.01 – 0.01	0.709
prior.density.cs	−0.04	−0.07 –−0.02	0.001	−0.02	−0.03 –−0.01	<0.001
density.cds	0.17	0.13 – 0.22	<0.001	0.19	0.15 – 0.22	<0.001
prior.density.cds	0	−0.05 – 0.05	0.888	0.05	0.01 – 0.10	0.011
**Random Effects**						
σ2	0.02			0.03		
τ00	0.02 name			0.02 name		
ICC	0.44			0.42		
*N*	147 name			109 name		
Observations	841			541		
Marg.*R*^2^/Cond.*R*^2^	0.668/0.813			0.595/0.767		

#### CS Node Eigenvector Centrality

Table 8 shows the results for the linear mixed models for both children, predicting CS eigenvector centrality. Each model is detailed below.

##### CS Centrality: Child 1

The model's total explanatory power is substantial (conditional *R*^2^ = 0.46) and the part related to the fixed effects alone (marginal *R*^2^) is of 0.44. Within this model, the following variables have a significant effect on CS1 centrality level: age (negative effect), CDS centrality (positive effect), CS centrality in recording N-1 (positive effect), CDS centrality in recording N-1 (positive effect), CS degree (positive effect), and CS density (positive effect).

##### CS Centrality: Child 2

The model's total explanatory power is substantial (conditional *R*^2^ = 0.43) and the part related to the fixed effects alone (marginal *R*^2^) is of 0.43. Within this model, the following variables have a significant effect on CS1 centrality level: age (negative effect), CDS centrality (positive effect), CS degree (positive effect), CS density (positive effect), and CDS density (negative effect).

#### CDS Centrality

Table 9 shows the results for the linear mixed models for both parents, predicting CDS eigenvector centrality. Each model is detailed below.

##### CDS Centrality: Child 1

The model's total explanatory power is substantial (conditional *R*^2^ = 0.81) and the part related to the fixed effects alone (marginal *R*^2^) is of 0.67. Within this model, the following variables have a significant effects on CDS1 centrality: CS centrality (positive effect), CDS centrality in recording N-1 (positive effect), CS degree in recording N-1 (positive effect), CDS degree (positive effect), CS density (positive effect), CS density in recording N-1 (negative effect), and CDS density (positive effect).

##### CDS Centrality: Child 2

The model's total explanatory power is substantial (conditional *R*^2^ = 0.77) and the part related to the fixed effects alone (marginal *R*^2^) is of 0.60. Within this model, the following variables have a significant effects on CDS2 centrality: age (negative effect), CS centrality (positive effect), CS centrality in recording N-1 (positive effect), CDS centrality in recording N-1 (positive effect), CDS degree (positive effect), CDS degree in recording N-1 (positive effect), CS density in recording N-1 (negative effect), CDS density (positive effect), and CDS density in recording N-1 (positive effect).

### Network Density

**Figure 6:**
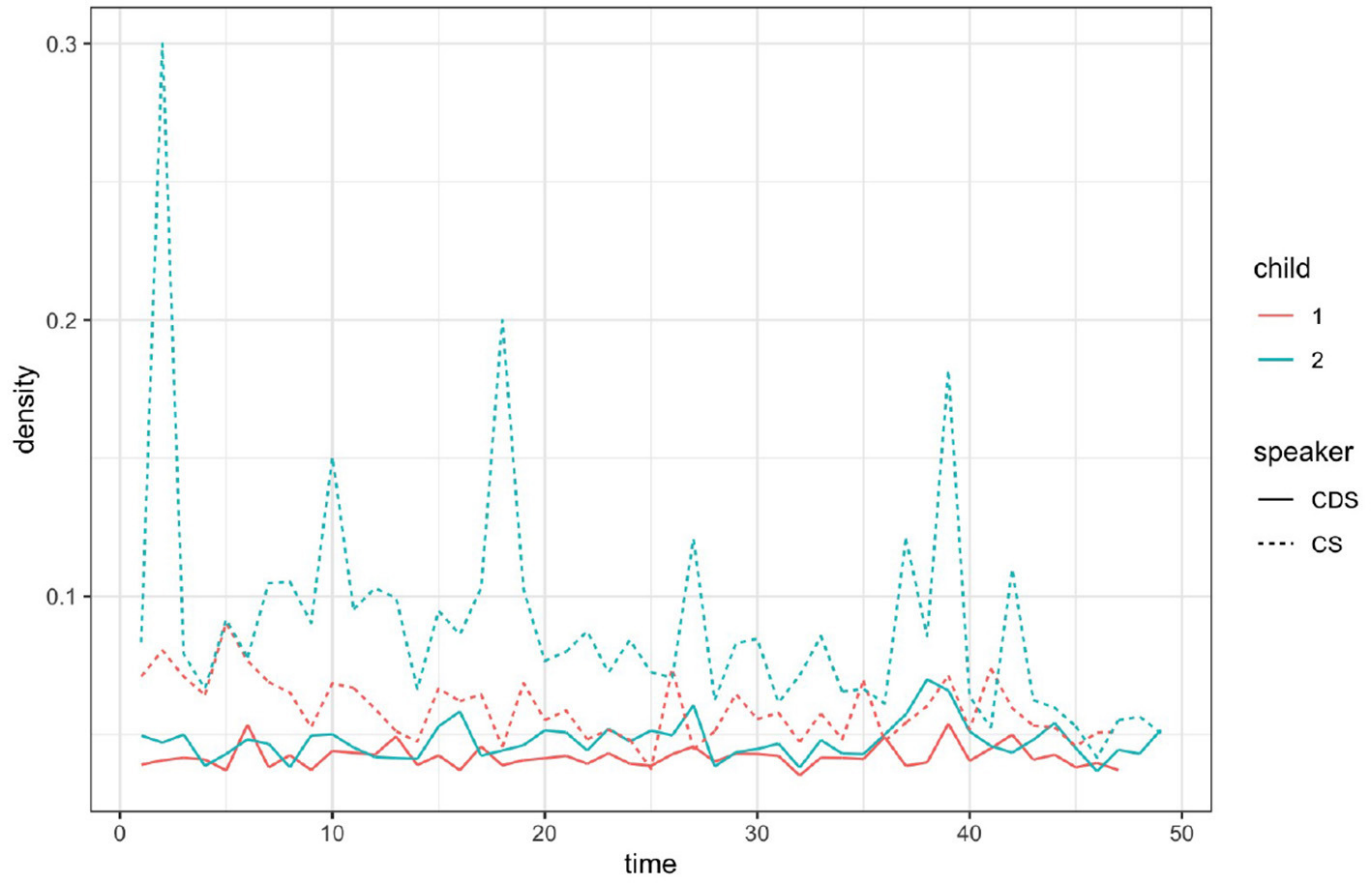
Network density by session, Child 1 and 2, CS and CDS. CS of Child 1 is represented by a dashed red line; CS of Child 2 is represented by a dashed green line; CDS of Child 1 is represented by a solid red line; CDS of Child 2 is represented by a solid green line.

Recall that network density measures the proportion of active links relative to the maximum number of possible links in the current network, given the active nodes. Figure 6 depicts the changing densities of networks with age for each of the participants.

Network density seems to decrease with age for both children (although at different rates), while it seems to remain constant for the parents through development. That is, as children grow, they exhaust fewer of their possible links, leaving more room for their network of roots and inflected patterns to grow: a non-exhausted network (i.e., a low density network) is a network with a high growth potential, since new links can be created between existing constructs that have not been linked before.

For each network we fitted a linear model to predict network density with the following variables: age, network density of the other party in the dyad, and network density at the preceding recording for both parties. As network density is a global measure relevant for the entire network, we did not include measures of individual nodes in these models (unlike the models presented above for degree and centrality).

#### CS Density

Table 10 shows the results for the linear models for both children, predicting CS network density. Each model is detailed below.

**Table 10:** Linear model: CS Network density.

**Predictors**	**density.cs: child 1**		***p***	**density.cs: child 2**		***p***
	**Estimates**	**CI**		**Estimates**	**CI**	
(Intercept)	1.31	1.07 – 1.55	<0.001	5.36	4.88 – 5.84	<0.001
age	−0.02	−0.03 –−0.02	<0.001	−0.1	−0.11 –−0.09	<0.001
density.cds	0.51	0.33 – 0.69	<0.001	0.92	0.57 – 1.27	<0.001
prior.density.cs	0.03	−0.07 – 0.14	0.544	−0.14	−0.23 –−0.06	0.001
prior.density.cds	−0.45	−0.64 –−0.25	<0.001	0.76	0.38 – 1.15	<0.001
Observations	841			541		
*R*^2^/*R*^2^ adjusted	0.276/0.273			0.397/0.392		

##### CS Density: Child 1

The model's explanatory power is substantial (*R*^2^ = 0.28, adj. *R*^2^ = 0.27). Within this model, the following variables have a significant effect on CS1 network density: age (negative effect), CDS density (positive effect), and CDS density in recording N-1 (negative effect).

##### CS Density: Child 2

The model's explanatory power is substantial (*R*^2^ = 0.40, adj. *R*^2^ = 0.39). Within this model, the following variables have a significant effect on CS2 network density: age (negative effect), CDS density (positive effect), CS density in recording N-1 (negative effect), and CDS density in recording N-1 (positive effect).

#### CDS Density

Table 11 shows the results for the linear models for both parents, predicting CDS network density. Each model is detailed below.

**Table 11:** Linear model: CDS Network density.

**Predictors**	**density.cds: child 1**		***p***	**density.cds: child 2**		***p***
	**Estimates**	**CI**		**Estimates**	**CI**	
(Intercept)	−0.95	−1.01 –−0.88	<0.001	−0.49	−0.64 –−0.34	<0.001
age	0.01	0.00 – 0.01	<0.001	0.01	0.00 – 0.01	0.001
density.cs	0.07	0.04 – 0.09	<0.001	0.05	0.03 – 0.07	<0.001
prior.density.cds	−0.36	−0.43 –−0.29	<0.001	−0.07	−0.16 – 0.02	0.141
prior.density.cs	0.27	0.24 – 0.31	<0.001	0.04	0.02 – 0.06	<0.001
Observations	841			541		
*R*^2^ / *R*^2^ adjusted	0.279/0.275			0.078/0.071		

##### CDS Density: Child 1

The model's explanatory power is substantial (*R*^2^ = 0.28, adj. *R*^2^ = 0.28). Within this model, the following variables have a significant effect on CDS1 network density: age (positive effect), CS density (positive effect), CDS density in recording N-1 (negative effect), and CS density in recording N-1 (positive effect).

##### CDS Density: Child 2

The model's explanatory power is weak (*R*^2^ = 0.08, adj. *R*^2^ = 0.07). Within this model, the following variables have a significant effect on CDS2 network density: age (positive effect), CS density (positive effect), and CS density in recording N-1 (positive effect).

### Interim Summary

The results reported above show that Hebrew verb morphology can be conceptualized as a network linking roots and patterns. This construal sheds new light on the development of this system with respect to patterns of adaptation within and between CS and CDS, as well as tracking small, but meaningful, changes within the system's structure. Looking at node activation in the network, we show that development is punctuated in terms of verb usage. The node degree measure reveals that the CS linkage level (of each root to number of patterns and each pattern to number of roots) is affected by the following factors: age; the CDS linkage level of the same root or pattern; the linkage level the same root or pattern had in the previous recording in CS; the root's or pattern's centrality within the network; and the network's density. The CDS degree (linkage level) is not shown to be affected by age, remaining steady throughout the time range of our data. It is, however, affected by the following: the CS linkage level and by the linkage level of the same pattern/root in previous recordings; the centrality of the pattern/root within the system; the density of the current network; and the density of previous networks of both the child and the parent.

The node centrality measure reveals that within the CS networks there are more central roots/patterns compared with the parents' networks. The centrality of a root/pattern within the CS morphological system (i.e., its centrality within the network) is affected by the following factors: age; the centrality of the same root/pattern in the CDS network; its degree in the CS network; and the CS network's density. The node centrality in the CDS network is not affected by age, but rather by the following: the previous centrality within the CDS network; the degree of the same root/pattern in the CDS network; the density of the CDS network; and the density of the previous CS network.

Finally, the network density measure reveals that CS network density is affected by age as well as by the density of the CDS—of both the previous and the current network. The CDS network density is affected by age as well (contra to the degree and centrality measures) and by the density of the CS network—both the previous and the current. In the following section we discuss each of these results and its implications in detail.

## Discussion

Recent studies on Hebrew verb acquisition (Ashkenazi et al., 2016, 2020) have shown that toddlers rely on stable, frequently occurring inflectional verb affixes in maternal input to gain salient information on the opaque, irregular verbs they frequently encounter. Furthermore, children's output greatly resembles and correlates with parental input in terms of structural, semantic and pragmatic features of the verbs used, highlighting the role of CDS in shaping CS verb structure. Previous studies indicated the possible contribution of CDS to CS verb content in the form of parental corrections, reformulations and expansions, children's uptake and imitations in parent-child conversations characterized by mutual attention and responsiveness (Clark and de Marneffe, 2012).

The present study adds to this line of research by accounting for the development of the morphology of Hebrew verbs as a dynamic network of roots and inflected patterns within the interactive domain of Child Speech and Child Directed Speech. The results presented in Figures 2–6 and the models in Tables 6–11 show that the development of the system of roots and inflected patterns between 1;8 and 2;2 is not strictly linear: it is not only a matter of mere frequency of use, but rather that across development, the child links more roots to every available pattern and inflection, and more patterns and inflections to every available root. Development is shown here to be dynamic, complex, and adaptive in several ways.

First, we saw that morphological development in children can be characterized as punctuated rather than continuous. Except for a few frequent nodes, most morphological constructs (i.e., roots and patterns) become active for a specific period of time, and then stop being used, sometimes re-appearing again in later periods. Conversely, as shown in Figure 3, the parent's use of morphological constructs is more coherent or continuous, such that each construct is used for a longer period of time, and breaks between uses are shorter. That is, it seems like the child is busy mastering each construct for a certain period, only to move on to another construct. Consider for example the CS1 data (CS of Child 1; top left panel in Figure 3): Node number 74 in the CS1 data is the pattern *Pi'el*.Inf; it is active at the first recordings, and then goes inactive and active again throughout the data. Conversely, node number 121 in the same data is the pattern *Qal*.Fut.3.Sg.Ms, and it is active in each and every recording.

Second, the models presented above reveal the dynamic nature of development, underscoring the role of adaptation—between the child and the parent, and relative to past experience. This is explained in the following sections, where we discuss (1) linkage (i.e., node degree) between roots and inflected patterns; (2) importance (eigenvector centrality) of roots and inflected patterns; and (3) systematic growth potential in the network (network density). Since we are interested in the dynamic nature of development through time, we included the level of the various measures at the prior recording as a possible explanatory variable for the measures of a current recording for both CS and CDS.

### Root and Inflected Pattern Degree Across Development

The degree of a node in a network measures the number of links it has with other nodes. In our case, links between nodes are verb wordforms created by the affixation of roots to inflected patterns. For example, the inflected pattern node *Qal*.Present.Fm.Sg, has a degree level of 4 in the CS network of Child 2, recording number 3, linked to the roots *r-*ʔ*-y, k-*ʔ*-b, n-w-*ℏ, and *b-w-*ʔ, manifested as the verbforms *ro'a* “sees/ing,” *ko'évet* “hurts/ing,” *náxa* “rests/ing,” and *bá'a* “comes/ing,” respectively. The same inflected pattern is expanded through development, having a degree level of 8 in recording number 32. This means that this inflected pattern is used in a larger grammatical network, linking other roots to the morphological family of the pattern. That is, it highlights another context in which this pattern can be used in terms of referents and event attributes.

The results of our models show that changes in the degree of roots and inflected patterns are not only a factor of age, but rather that the degree level of a morphological construct is systematically related to other factors within the network and within the dyad. These results are different for the CS and the CDS, and the two models are presented separately. We discuss only those results that were significant for both children, leaving individual differences to future research.

Results for the CS degree models (Table 6) show that the number of roots predicted to be linked to a single inflected pattern and the number of inflected patterns a single root is linked to are affected by the age of the child, such that with age, each construct is predicted to be linked to more constructs. The linkage level is also affected by the number of links these constructs have in the previous network of the child speech, and by the number of links they have in the parent's current network. The importance of these constructs in the child's speech also affects their linkage, such that more central nodes are predicted to have more links. Note that the centrality of the nodes in the parent's network does not have a significant effect on the child's degree. That is, for a construct to have more links in the CS, it is crucial that it has more links in the CDS, but not that it has a prominent position in the CDS network. Finally, another factor that is common to both children is the effect of the CS network density on degree level, such that degree levels decrease with higher network density. This is a manifestation of the growth potential interpretation of the density measure, proposed by Levie et al. (2019), since lower density levels indicate a higher potential for the network to grow, realized here as higher degree levels.

Results for the CDS degree model (Table 7) show that age has differential effects in the two children participating in the current study: while the degree levels in parental speech rise in the CDS of Child 1, they fall in the CDS of Child 2. However, the degree level in the current CS network and degree level in the previous CDS network have the same effect in both parents: increasing degree levels in the current network of the CS and in the previous CDS network predict increase in the degree levels in the CDS. That is, linkage between roots and inflected patterns within the parent's speech is affected by the current speech of the child, as well as the previous speech of the parents themselves. We may conclude that parents adapt to their children's speech in two ways: first, by relating to their children's current usage. Second, by expanding on previous experience, counting on the usage their children have already been exposed to and building upon it. Interestingly, degree levels in the previous recording in the CS do not have an effect on current degree in the CDS. That is, parents do not build on their children's previous usage, but on their own. This effect, too, should be further investigated in future research.

The importance of nodes in the current network (as realized by eigenvector centrality) affects CDS linkage as well, such that more important nodes are predicted to have more links. Note that this applies only to the importance of the nodes in the current network of the parents, but not to the current network of the child nor to previous networks of the child or the parent. That is, in contrast to previous linkage, which seems to affect current linkage in both children and parents, the importance of morphological constructs has an effect only on the current network. This may be explained by the fact that centrality (i.e., importance) is a more context specific measure than degree (i.e., linkage), and context is changing from one recording session to another. Linkage, however, is a matter of morphological productivity, linking a single root to relatively many inflected patterns, and vice versa. Therefore, it is a systematic measure that grows incrementally.

### Root and Pattern Centrality Across Development

The eigenvector centrality of a node is a measure of its importance within the network: It assigns a value to a node based on the number of links it has with other nodes that have many links themselves. For example, in our case, a *binyan*-temporal pattern that is linked to many roots that are linked to other *binyan*-temporal patterns has high centrality. While the measure of degree discussed above underscored the importance of linkage in expanding the network, eigenvector centrality is a relative measure, highlighting network variability. A network with few highly centralized nodes has few hubs through which information in the network can flow. In morphological terms we can think of it as a network with a small number of inflected patterns that are linked to many roots, each of which is linked to other patterns as well. This type of network limits the possibility to link a root to an inflected pattern that is not central in the network: in a given conversation, it is more probable for a new link to be made between a root and a central inflected pattern than with a less central one (resembling diversity situations with high entropy). However, if the network has many low centralized nodes, with no single node (or few nodes) standing out from the crowd, the probability of making new links is higher. Figure 5 shows that this is indeed the main difference in centrality distribution between the CDS and CS of both dyads in our data.

Results for the CS centrality model (Table 8) show that the eigenvector centrality of a root or an inflected pattern is predicted to fall with age. We interpret this result as indicating that networks become more evenly distributed in terms of centrality with age, thus enabling morphological productivity in language use: Since there are no few nodes that stand out from the crowd, the probability to use each node in the network (rather than just a few) is increasing (resembling a low entropy system). Thus, the structure of the system not only reflects productivity, but rather enables it, as more nodes gain usage probability. The centrality of a node in the children's networks is also affected by the centrality of this node in the parent's network, such that rising CDS centrality predicts rising CS centrality. That is, if on a given day a root is used with many patterns that are used with many roots in the parents' speech, than this root is predicted to be used with many patterns that are used with many roots in the children's speech as well. The degree of a node also affects the centrality of the node, but only relative to its linkage in the children's speech. The degree of a node in the parents' speech does not predict its centrality level in CS. Finally, the density of the network has an effect as well, such that rise in density levels (i.e., a less varied network) predicts high centrality. That is, density, as a growth potential measure, affects not only local morphological productivity (as seen above in the discussion of the degree as morphological linkage), but also systematic morphological productivity, such that a network with more growth potential is a less centralized system.

Results for the CDS degree model (Table 9) show that eigenvector centrality of roots and inflected patterns in the parents' speech to children is not affected by the age of the child. That is, the system is stable over time in terms of construct importance. However, while it is stable as a system, the network is still adaptive at a more local level: the centrality of roots and inflected patterns in the CDS changes as a function of their centrality in the CS. That is, parents adapt the particular morphological construct they use to the usage of their children, putting the burden of morphological productivity on the same constructs their children already use. The model also shows an effect for the centrality in the previous CDS network on current centrality. We interpret this result as indicating continuation and coherence: If a morphological construct is important in the network, a parent will keep using it in an important manner. This may facilitate learning, as it provides the child with more learning opportunities with the same distributional characteristics. The effect of CDS density on CDS centrality is similar to the effect of CS density on CS centrality discussed above: High CDS density predicts a more centralized network, interpreted as a less variable and less productive one.

Finally, adaptation can also be seen in the effect of CS density in the prior network on CDS centrality, an effect that was absent in the CS model for centrality. The CDS centrality model shows that a rise in density levels in the CS previous network predicts a fall in centrality levels in the CDS current network. High density and low centrality can be thought of as two sides of the same coin, denoting systematic productivity. That is, a network with high density has low growth potential since most of its possible links have already been made. Conversely, a system with many low central nodes is less limited in its potential to form new links, as probability is more evenly distributed. The current model shows that if the previous network of the children had low growth potential, the current network of the parents is systematically more productive. This can be seen as fine-tuned tweaking of the system toward productivity by the parent: when parents experience a limited network in the speech of their children, they will provide them with more opportunities to expand their system in future interactions.

### Morphological Network Density Across Development

The density of the network is a measure of fulfilled links among nodes, relative to all possible links. As such, this measure quantifies the level of network exhaustion in terms of how much of the potential of the current network has already actually been fulfilled by the speaker. A speaker that has already fulfilled most of her network's entire potential has nowhere to grow, in the sense that the probability of re-using existing verb wordforms (i.e., links within the networks) is high. In such a network, the main road to expansion is by adding new nodes, and not by creating new links. Thus, a child with a high density network needs to add more roots and/or inflected patterns to her network in order to expand her morphological verb lexicon. On the other hand, a child with a low density network can expand her network also by linking constructs that were not linked before, creating variations on verb wordforms.

Figure 6 and the models summarized in Tables 10, 11 show that for both children, CS network density is affected by the child's age, such that networks become less dense with age. CDS network density is affected by age in the opposite direction, with density rising with age. Patterns of adaptation are manifested in the relations revealed here between the density of the CS networks and that of the CDS networks: CDS network density affects CS network density for both children, such that children's network density is adaptive to that of their parents: the higher the density of the CDS network, the higher the density of the CS network. Parents are also adaptive to their children's network density: CDS network density is affected by CS network density, such that higher CS density levels predict higher CDS density levels. The model also shows that CDS network density is affected by the density of the CS in the previous recording.

These results demonstrate the manifestation of adaptation: both parties in the dyad adapt their network structure to that of their interlocutor, taking into account their current and the previous states (with some individual differences). Going from the statistics to morphology, we interpret density as growth potential (Levie et al., 2019). Thus, we can conclude that the potential to expand the network, forming new links between existing roots and inflected patterns, is a function of more than just the age of the child, with the current and previous morphological network structures of the other party in the developmental tango also having an effect.

An illuminating step in the development of a morphological system is morphological over-generalizations. However, in the present study we do not report on such morphological errors, that are the result of linking an existing root to an existing pattern within the network, creating a link that is not observed in the adult language. The fact that we did not find such errors might seem surprising, but we believe it is due to the nature of our particular data. Specifically, we claim that the age range of our data (1;8–2;2) might be too early for morphological over-generalizations of this type in Hebrew. We might find errors in this age range in terms of word order or agreement marking, but derivational root-*binyan* errors are more typical of children aged 4 years and above, indicating the consolidation of verb morphology (Levie et al., 2020). We assume that recordings of older child-adult dyads may reveal more over-generalizations, since the morphological system would gain more network growth potential, as shown above, until reaching a point of equilibrium in terms of network density, balancing between creativity and conventionality (Tomasello, 2000).

### Converging Models

**Figure 7:**
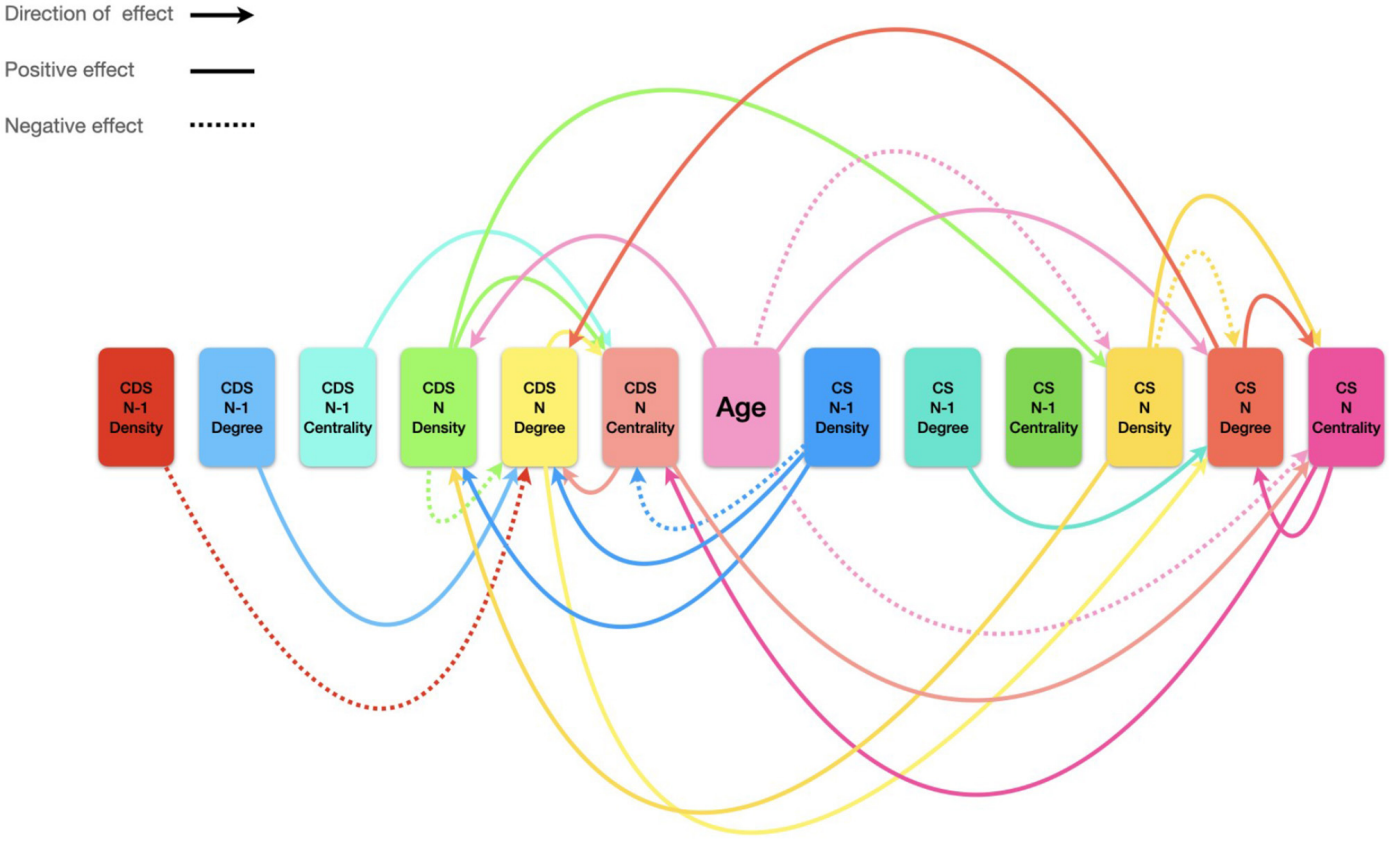
A developmental model of a morphological verb lexicon network: The image shows a dynamic system of adaptation involving the child, the parent, and time. Arrows between affecting and affected network measures are colored according to the affecting variable; solid lines indicate positive effects, and dashed lines indicate negative effects.

Figure 7 is a visual summary of all significant effects found in the models discussed above, suggesting a complex unified model for patterns of adaptation in Hebrew verb morphology development between the ages of 1;8–2;2 as a network.

Figure 7 shows that many of the variables both affect and are affected by other variables, within the speaker (child or parent), and between speakers. This indicates dynamic relations within the changing system of the morphological verb lexicon involving adaptation within the speaker, between the speakers, and relative to past experience.

## Conclusion

Language learning is dynamic (van Geert, 1991, 2010), changing as a function of age, individual differences, and input language (CDS), among other factors. Within this growing/emerging system, CDS and CS affect each other in different ways. The current paper shows these relations, modeling the development of Hebrew verb morphology between the ages of 1;8–2;2.

We used network analysis to account for complex relations between morphological constructs, so as to assess changes in network structure over time. Growth in frequency during development is unquestionably apparent. With age, children are shown to produce more roots, more inflected patterns, and more wordforms. Using network analysis we also showed that children's networks are growing with age as well, in terms of node degree and centrality representing linkage level and construct importance respectively, and in terms of the network density as representing network growth potential. However, this method allowed us to go beyond growth to the crucial role of variation, showing that both take part in development. A major finding reported above is that development is not linear, and that children go through periods of punctuated development: We saw that children's use of morphological constructs is not coherent, in the sense that it is not the case that once a child is using a construct she will continue to use it in a productive manner. Rather, the children were shown to use individual constructs for short periods of time. This finding is highlighted by contrasting it with the parents' patterns of usage, which is much more coherent or continuous. This leads us to the conclusion that children between the ages of 1;8–2;2 do not use their entire range of possible constructs based on a cumulative lexicon. Rather, children are attuned to their immediate experience: If a morphological construct was highly linked or very important in the immediate past, or if it is important within the network of their caregiver, it is more likely to be used again by the child. This expresses the variation of the developing dynamic system. The productivity of morphological constructs is varied, and depends on many different (and related) factors.

Another facet of dynamic development revealed by the network analysis concerns the adaptation of the parents to their children's systems. We show that parents adapt to their children's speech patterns in three ways: first, by relating to their children's current usage. Second, by expanding on previous experience, counting on the usage their children have already been exposed to, and building upon it. And third, we show that when parents experience a limited network in the speech of their children, they will provide them with more opportunities to expand their system in future interactions.

A dynamic system goes through changes that are a function of its current state (van Geert, 2010). The analysis suggested in the present paper shows that this is an apt description of the development of Hebrew verb morphology, and that the framework of dynamic network analysis thus provides insights into the complex issue of language development. Given that, we would like to point out two directions for future research. First, the present paper modeled each variable in a separate manner, while the converged model presented in Figure 7 suggests that an improved model might arise by analyzing the entire array of variables simultaneously so as to expose interactions. Second, the discussion of the results in the present paper focused on those cases where no individual differences were found. As shown in the summaries of the models, there are individual differences between the two children and the two parents in the current database. This suggests that analyzing such data by taking not only the item itself as a random variable (as done in the present paper), but also the speaker, and expanding our sample, might reveal even more interesting relations within the dynamic system.

## Data Availability Statement

The datasets presented in this article are not readily available because it's identifiable data. Requests to access the datasets should be directed to Orit Ashkenazi, orit.ashkenazi@gmail.com.

## Ethics Statement

Ethical review and approval was not required for the study on human participants in accordance with the local legislation and institutional requirements. Written informed consent to participate in this study was provided by the participants' legal guardian/next of kin.

## Author Contributions

ED wrote most of the paper, conceptualized the research questions and the method of analysis, conducted the analyses, and provided most of the insights. RL conceptualized and carried out the framework for verb analysis and provided analyses of the data and insights. DR wrote the morphological parts of the paper, participated in conceptualizing the interpretation of the results, and edited the paper. OA collected and analyzed the database, wrote the input-output section, and provided insights. All authors contributed to the article and approved the submitted version.

## Funding

This study was supported by ISF grant no. 219/17 to DR.

## Conflict of Interest

The authors declare that the research was conducted in the absence of any commercial or financial relationships that could be construed as a potential conflict of interest.

## Publisher's Note

All claims expressed in this article are solely those of the authors and do not necessarily represent those of their affiliated organizations, or those of the publisher, the editors and the reviewers. Any product that may be evaluated in this article, or claim that may be made by its manufacturer, is not guaranteed or endorsed by the publisher.

## Footnotes

^1^Roots are represented as morphological entities, that is, taking into account their morpho-phonological behavior, as detailed in Ravid (2012). For example, the *k* phoneme in the root *k-t-b* alternates with spirant *x* phoneme, while the *k* phoneme in *q-d-m* does not (Temkin Martinez, 2010). For words (in contrast to roots) we use a broad phonemic transcription.
